# The Effect of Teriparatide on Fracture Healing of Osteoporotic Patients: A Meta-Analysis of Randomized Controlled Trials

**DOI:** 10.1155/2016/6040379

**Published:** 2016-06-26

**Authors:** Shenghan Lou, Houchen Lv, Guoqi Wang, Licheng Zhang, Ming Li, Zhirui Li, Lihai Zhang, Peifu Tang

**Affiliations:** Department of Orthopedics, General Hospital of Chinese PLA, Beijing, China

## Abstract

*Purpose.* This meta-analysis is to assess the effectiveness of teriparatide in fracture healing and clinical function improvement of the osteoporotic patients.* Methods.* We searched PubMed, Embase, Web of Science, and the Cochrane databases for randomized and quasi-randomized controlled trials comparing teriparatide to placebo, no treatment, or comparator interventions in the osteoporotic patients.* Results.* Five studies with 251 patients were included. Patients treated with teriparatide therapy had a significant shorter radiological fracture healing time compared with those in the control group (mean difference [MD] −4.54 days, 95% confidence interval [CI] −8.80 to −0.28). Stratified analysis showed that lower limb group had significant shorter healing time (MD −6.24 days, 95% CI −7.20 to −5.29), but upper limb group did not (MD −1 days, 95% CI −2.02 to 0.2). Patients treated with teriparatide therapy showed better functional outcome than those in the control group (standardized mean difference [SMD] −1.02, 95% CI −1.81 to −0.22). Patients with therapy duration over 4 weeks would have better functional outcome (SMD −1.68, 95% CI −2.07 to −1.29).* Conclusions.* Teriparatide is effective in accelerating fracture healing and improving functional outcome of osteoporotic women. However, more clinical studies are warranted in order to determine whether the results are applicable to males and the clinical indications for teriparatide after osteoporotic fractures.

## 1. Introduction

Bisphosphonates, the synthetic analogues of pyrophosphate [1], are the most widely used medications for the treatment of osteoporosis [2, 3]. The mechanism of bisphosphonates is that bisphosphonates accumulate in bone by binding to mineral crystals and bisphosphonates like alendronate preferentially deposit, not in newly formed bone, but beneath osteoclasts. The key pharmacological action of bisphosphonates is the inhibition of osteoclast-mediated bone resorption. Bisphosphonates inhibit the formation and aggregation of calcium phosphate crystals to prevent bone loss and improve bone strength [4]. However, a long-term use of bisphosphonates may decrease bone formation, which is attributed to the long-term inhibition of osteoclasts [5]. Osteoclasts play an important role in remodeling of the callus into cortical bone. Thus, bisphosphonates may produce adverse effects on the healing process of fractures [6, 7].

Parathyroid hormone (PTH), an 84-amino acid peptide secreted by the parathyroid gland, is an important systemic regulator of calcium homeostasis [8]. It has been demonstrated that intermittent administration of PTH leads to an anabolic effect on bone [9]. Teriparatide, a synthetic polypeptide hormone consisting of the 1–34 fragment of human parathyroid hormone, retains most of the biological activities of PTH [10]. Intermittent administration of teriparatide also has the anabolic effects that stimulate bone formation and activate bone remodeling [11], improving the microarchitecture of trabecular bone and cortical bone [12]. Previous studies reported that teriparatide also increased bone mineral density and decreased risk of vertebral and nonvertebral fractures [13–16]. Meanwhile, teriparatide was the only anabolic drug for osteoporosis adopted by the Food and Drug Administration [17].

Previous studies reported favourable changes in bone mineral content, structure, and microarchitecture after teriparatide treatment due to the anabolic effect of this drug [18, 19]. As intermittent administration of teriparatide can stimulate bone formation, it seems reasonable to assume that teriparatide might accelerate fracture healing simultaneously in osteoporosis cases with fractures [20–23]. Recently, some studies about osteoporotic fracture cases indicated a beneficial effect of teriparatide [24–26], but the latest two randomized controlled trials (RCTs) showed that there were no statistically significant differences between the group treated with teriparatide and the group treated with placebo [27, 28]. As evidence-based evaluation of this issue is limited, the effect of teriparatide on osteoporotic fracture healing remains controversial [29].

Therefore, to determine whether teriparatide accelerates fracture healing in the osteoporotic patients, we performed a meta-analysis of RCTs. In this study, we evaluated the effectiveness of teriparatide in osteoporotic fracture healing and clinical function improvement. The results of this study would elucidate whether teriparatide would be effective in inducing fracture healing and improving functional outcome for the osteoporotic fracture patients.

## 2. Material and Methods

### 2.1. Search Strategy

A search of PubMed, the Cochrane Library, and Embase was performed in November 2015 for studies published between 1966 and October 2015, using the following combination of terms: “teriparatide” or “Parathyroid Hormone” or “forteo” or “PTH (1–34)” or “PTH (1–84)” or “parathormone” or “parathyrin” and “fractures healing” or “healing” and “fractures, bone” or “broken bone” or “bone fracture” or “fractures.” Google Scholar was also used to screen relevant literature, and the reference list was manually searched from all the relevant original research and review articles to identify additional potentially eligible studies. There were no language restrictions on trial eligibility.

### 2.2. Selection Criteria

Studies were included if they met the following criteria: (1) study design was a RCT; (2) participants had osteoporosis with fractures, and (3) the intervention was teriparatide initiation compared with placebo, no treatment control group, or comparator interventions, such as vitamin D, bisphosphonates, analgesics, and calcium.

Studies were excluded if they met the following criteria: (1) participants younger than 50 years of age, (2) contraindication to any of the study drugs, formerly or currently on any of them, (3) serum calcium above the reference level and liver enzymes more than double of the upper reference level, (4) history of tumor or chemotherapy, bone metastases, open or pathologic fractures, known metabolic bone disease, rheumatoid arthritis or chronic renal failure, joint disease, or any disease affecting bone metabolism; (5) the articles which were not available or had repeated data.

### 2.3. Data Collection and Endpoints

The appropriate articles were verified by two independent investigators (S. Lou and G. Wang). In case of disagreement between the two investigators, a third one was consulted. We extracted information of the participants' characteristics, type of fracture and treatment, time of teriparatide initiation and treatment, follow-up, time of radiological fracture healing, and functional outcome from each study.

The primary endpoint was the time of fracture healing, as determined by radiography, which was defined as the time of cortical bridging in three of four cortices. The functional outcome was defined as an improvement in mobility at week 12 and assessed with the Timed “Up and Go” (TUG) test or the self-administered “Patient-Rated Wrist Evaluation” (PRWE) questionnaire or “disabilities of the arm, shoulder, and hand” (DASH) score or the “Johanson Hip Rating Questionnaire” (JHRQ) [27, 28, 30, 31].

### 2.4. Quality Assessment

The RCTs were evaluated by the “Cochrane Collaboration's tool for assessing the risk of bias,” which included the following aspects: (1) random-sequence generation (selection bias); (2) allocation concealment (selection bias); (3) blinding of participants and personnel (performance bias); (4) blinding of outcome assessment (detection bias); (5) incomplete outcome data (attrition bias); (6) selective reporting (reporting bias); (7) other bias.

### 2.5. Grading Quality of Evidence

Two authors (SHL, HCL) independently evaluated the quality of evidence for primary and secondary outcomes according to the Grading of Recommendations Assessment, Development, and Evaluation (GRADE) [32] methodology for risk of bias, inconsistency, indirectness, imprecision, and publication bias. The assessment results were classified as very low, low, moderate, or high. Summary tables were constructed using the GRADE Profiler (version 3.6).

### 2.6. Statistical Analysis

Statistical analyses were performed with Review Manager Software (version 5.3; the Nordic Cochrane Center, the Cochrane Collaboration, Copenhagen, Denmark). Continuous outcomes were expressed as mean difference (MD) and 95% CI. Dichotomous outcomes were expressed as OR and 95% CI. To assess heterogeneity in results of individual studies, we used Cochran's *Q* statistic, *I* ^2^ statistic (*I* ^2^ > 50% was used as a threshold indicating significant heterogeneity), and *P* values (*P* value < 0.10 was used as a threshold indicating significant heterogeneity) [33]. A fixed effects model was applied in the meta-analysis. However, a random effects model was used when significant heterogeneity was found [34]. In the planning stage, sensitivity analysis would be performed by omission of each study to evaluate stability of the results if heterogeneous studies existed. Funnel plots were used to assess for publication bias. All tests were two-tailed and *P* value < 0.05 was deemed statistically significant.

## 3. Results

### 3.1. Literature Search and Characteristics

**Figure 1:**
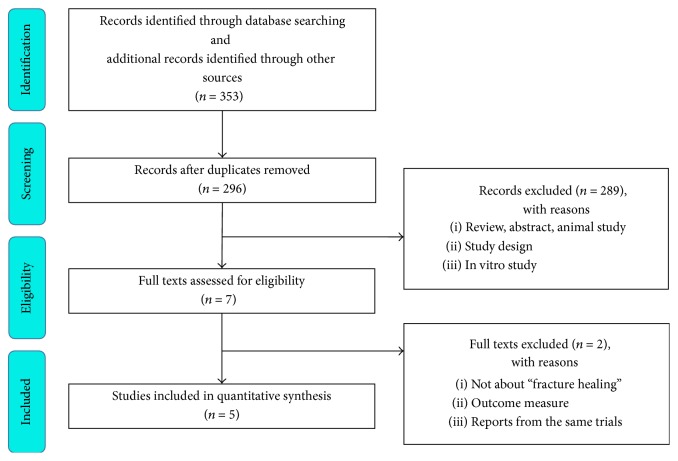
Flow diagram shows the process of literature selection.

Our search strategy identified 296 relevant articles, the titles and abstracts of which were screened for inclusion. The full text of 7 articles was retrieved, 5 of which [27, 28, 30, 31, 35] met the inclusion criteria. A manual search of the reference list within these studies did not yield any additional eligible studies.  Figure 1 illustrates the process of study selection.

A total of 251 patients were randomly assigned in the 5 trials included in this meta-analysis. There were 125 patients in the experimental group, and 126 in the control group. Regarding sex, 3.1% (*n* = 8) of the patients were male and 96.9% (*n* = 243) were female. The overall mean age was 70.3 years. As for the fracture type, two trials had upper limb fractures, including distal radial fractures and proximal humeral fractures, and three trials had lower limb fractures, including pelvic fractures and hip fractures. The detailed characteristics of the included studies are listed in Table 1.

The experimental group included teriparatide or PTH1–84. There were differences in pharmacokinetics and actions between these two kinds of PTH, which resulted in the anabolic effect of 100 *μ*g PTH1–84 being equal to 20 *μ*g teriparatide. The control group included placebo, no treatment, or other drugs interventions. The study of Kanakaris et al. [27] had two kinds of control groups, either only vitamin D and calcium or those plus bisphosphonate (Alendronate, 70 mg orally). Another study [31] had two kinds of experimental groups, among which 20 *μ*g or 40 *μ*g teriparatide was taken. Both of them were united as one in these two studies. The treatment time of teriparatide varied from 4 weeks to 24 months in the experimental group. The detailed characteristics are listed in Table 2.

### 3.2. Methodological Quality

**Figure 2:**
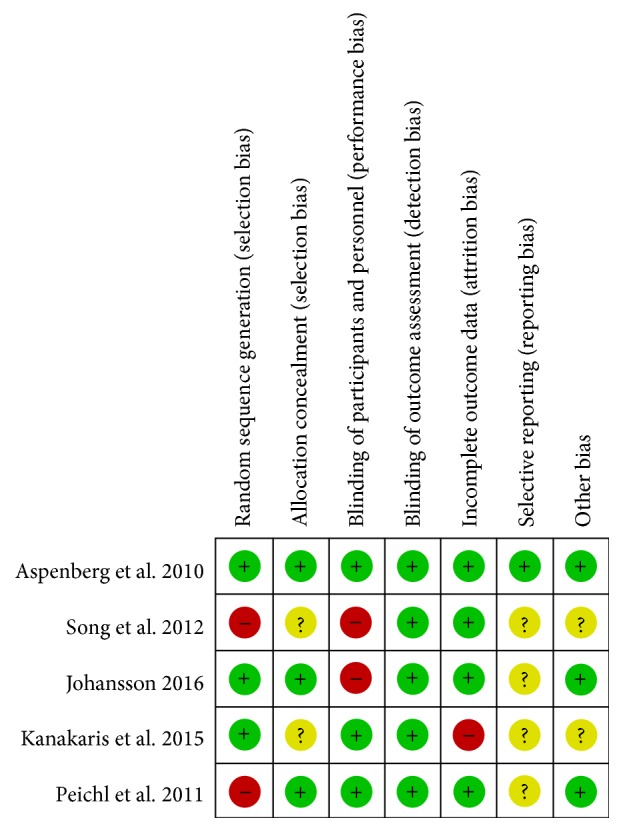
The methodological quality of the RCTs. Risk of bias summary. “+” means low risk; “?” means unclear risk; “−” means high risk.

The methodological quality of the RCTs is presented in Figure 2. The rate of patients lost to follow-up was appraised, and the dropout rate in only one trial was high (60%) [27].

### 3.3. Fracture Healing Time

**Figure 3:**
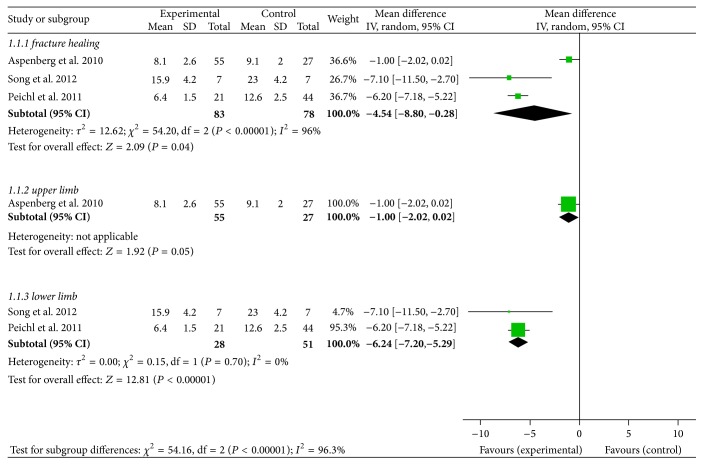
Forest plot for radiological fracture healing time.

The time of radiological fracture healing was defined as the time of cortical bridging in three of four cortices. Three trials met the inclusion criteria of this meta-analysis [30, 31, 35]. According to the results, patients who were treated with teriparatide had statistically significant difference in radiological fracture healing time compared with the control group (MD −4.54, 95% CI −8.80 to −0.28; *I* ^2^ of heterogeneity 96%, *P* < 0.00001; random effects model) (Figure 3). As *I* ^2^ = 96%, apparently over 50%, indicated significant heterogeneity, we further performed a sensitivity analysis and found that one trial [31] significantly affected the pooled MD. Therefore, a subgroup analysis, consisting of upper limb group (MD −1, 95% CI −2.02 to 0.2; *P* = 0.05; random effects model) and lower limb group (MD −6.24, 95% CI −7.20 to −5.29; *I* ^2^ of heterogeneity 0%, *P* = 0.70; random effects model), was performed (Figure 3). A visible difference was found between the upper limb and the lower limb.

### 3.4. Functional Outcome

**Figure 4:**
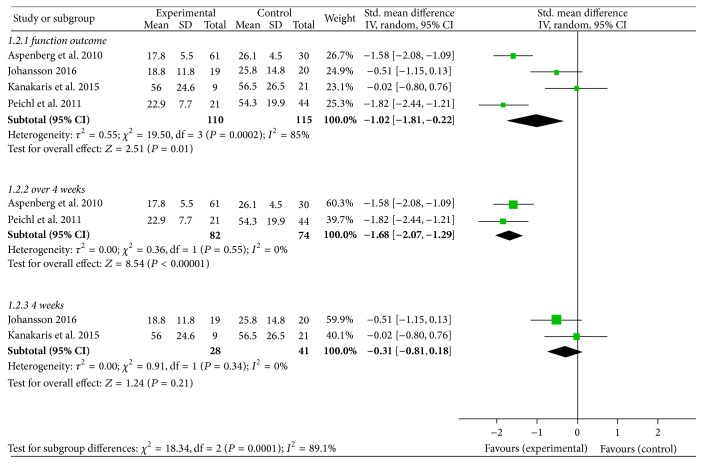
Forest plot for functional outcome.

The functional outcome was defined as an improvement in mobility at week 12 and assessed with the TUG test [30] or the self-administered PRWE questionnaire [31] or DASH score [28] or the JHRQ [27]. Because of the different measurement methods, a standardized mean difference method was used. Four trials were eligible for the meta-analysis of the functional outcome [27, 28, 30, 31]. Patients who were treated with teriparatide showed significantly better functional outcome than those in the control group (SMD −1.02, 95% CI −1.81 to −0.22; *I* ^2^ of heterogeneity 85%, *P* = 0.00002; random effects model) (Figure 4). In view of *I* ^2^ = 85% symbolized significant heterogeneity, we performed a subgroup analysis, in which one group represented that treatment time exceeded 4 weeks (SMD −1.68, 95% CI −2.07 to −1.29; *I* ^2^ of heterogeneity 0%, *P* = 0.55; random effects model) and the other represented that treatment time was equal to 4 weeks (SMD −0.31, 95% CI −0.81 to 0.18; *I* ^2^ of heterogeneity 0%, *P* = 0.34; random effects model) (Figure 4). The duration of treatment was the key factor for the function outcome.

### 3.5. Sensitivity Analysis

Sensitivity analysis was performed by omission of each study to evaluate stability of the results if heterogeneous studies existed. The sensitivity analysis for the fracture healing time showed that the study of Aspenberg et al. [31] significantly affected the pooled MD (Table 3). The sensitivity analysis for functional outcome showed that any study did not significantly affect the pooled MD (Table 4).

### 3.6. Publication Bias and GRADE Profile Evidence

**Figure 5:**
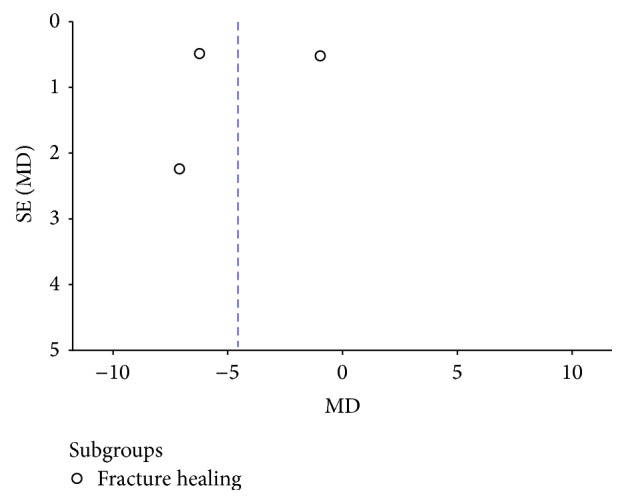
Funnel plot for fracture healing.

**Figure 6:**
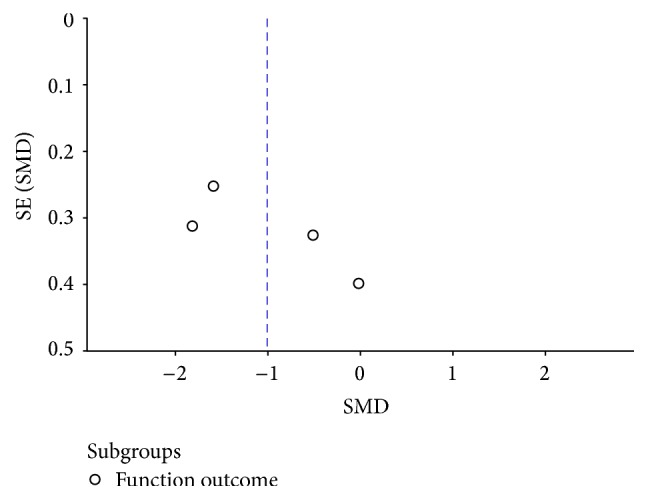
Funnel plot for functional outcome.

For the meta-analysis of fracture healing and functional outcome, there was no evidence showing obvious publication bias by examining the symmetry of the funnel plot (Figures 5 and 6).

GRADE evidence profiles for the primary and secondary outcomes were shown in Table 5. The most common reasons for the decreased level of evidence were the heterogeneity and suspected publication bias.

## 4. Discussion

### 4.1. Main Findings

As far as we know, this is the first meta-analysis to examine the effect of teriparatide on fracture healing and functional outcome of the osteoporotic patients. Our meta-analysis comprehensively and systematically reviewed the current available literature in regard to the teriparatide therapy for osteoporotic patients and found that (1) teriparatide therapy promoted osteoporotic fracture healing and the evidence of outcomes was confirmed by the GRADE system, although this evidence came from only three trials; (2) teriparatide therapy improved function outcome, which was confirmed by the GRADE system as well.

Furthermore, subgroup analysis showed that the effect of teriparatide was significantly related to the time of drug application and the site of fractures. Fractures healed by different mechanisms, depending on the location of fractures. One possible explanation is that the anabolic effect of teriparatide is further enhanced when bone is subjected to mechanical stimulation, and fractures of the load-bearing bone might be more susceptible to teriparatide. Similarly, compared with 4-week treatment [27, 28] or 8-week treatment [31], 24-month treatment [30] produced statistically significant results in terms of fracture healing and functional improvement. Although teriparatide could improve early callus formation [36], better effects may be seen with a longer duration of treatment.

#### 4.1.1. Implications for Clinical Practice

Fracture healing is a very complex process that involves both resorptive and formative processes. For osteoporotic patients, the long bone is ductile and shows plastic deformation before fractures. The main effect of teriparatide is to stimulate bone formation without stimulating bone resorption, which is called “anabolic window.” There are multiple mechanisms for teriparatide to promote fracture healing, including promoting proliferation and differentiation of mesenchymal stem cell, chondroprogenitors and osteoprogenitors, chondrocyte maturation, production of bone matrix proteins, and osteoclastogenesis. During fracture healing, it can increase callus formation by increasing the induction of proliferation and differentiation of osteoprogenitors and chondroprogenitors [37, 38]. It also enhances callus formation and callus remodeling by stimulating bone matrix protein synthesis and osteoclastogenesis [38]. In addition, teriparatide increases fracture callus size and expression of types II and X collagen via the Wnt/*β*-catenin signaling pathway [39].

The primary outcomes of our meta-analysis showed that, in case of osteoporotic fractures, teriparatide is a viable therapy that is not only able to treat the underlying osteoporosis but also able to accelerate fracture healing, especially for osteoporotic women. Correspondingly, it has been discovered that there are different effects between upper limbs and lower limbs via a subgroup analysis in our meta-analysis, and the lower limb group has a better result than the upper limb group.

To date, no systemic treatment is approved for fracture healing. Impaired healing of fractures delays the rehabilitation process, which influences life quality of the patients. At the same time, the associated costs cause an economic burden to both the society and the patients. Teriparatide therapy accelerates healing, which allows patients to return to normal life and work faster, and it reduces the medical consumption and chronic morbidity associated with long-term treatment. Furthermore, it can be applied to any types of fractures, including those that will be treated nonsurgically, can be commenced at any time, and can be applied through the entire healing period as well. As teriparatide therapy can promote osteoporotic fracture healing and improve function outcome, we suspect that teriparatide may prove to be useful in the stimulation of implant anchoring and fixation for both dental and orthopedic implants. Likewise, it may prove to be useful in fractures which have a high risk of delayed union or nonunion, as well as a very high degree of associated disability. Some studies have been started to explore related issues [40–45], but studies are still limited, and most of them are case reports. The hypotheses still need evaluation by high-quality randomized controlled trials.

#### 4.1.2. Strengths and Limitations

A major strength of this meta-analysis was compliance with the PRISMA guidelines and the recommendations of the Cochrane Collaboration, although our study was not registered with protocol. Additionally, the GRADE system was used to evaluate the quality of evidence for the outcomes in this meta-analysis.

There were, however, several limitations of this meta-analysis. First, only five articles were included, and sample size of the most included studies was small. Second, two studies [27, 31] contained multiple groups. We combined these groups together because the effect of teriparatide was studied in a broad sense, not compared with a particular medication. Third, publication bias was unclear due to the limited studies. Additionally, as more than 75% of osteoporotic fractures occurred in women [46] and only six patients of the study were males, the primary result seemed more applicable to osteoporotic women. Some more RCTs are needed to determine whether the results are applicable to males. Finally, in spite of adopting the proven methods [47] to estimate the missing data, a more detailed and comprehensive analysis was restricted.

## 5. Conclusion

There is no reason to believe that teriparatide will not be effective in improving functional outcome and inducing fracture healing in the osteoporotic women. However, researches in this field are not enough, some more clinical studies are needed to increase the quality of evidence and determine whether the results are applicable to males. Meanwhile, a number of clinical studies are warranted in order to determine the usefulness of teriparatide and the clinical indications for the use of teriparatide in the treatment of the osteoporotic fracture healing.

## Figures and Tables

**Table 1:** Characteristics of included studies.

Studies	Number of patients	Age/years		Type of fracture	Sex	
		Mean	SD		F	M
Kanakaris et al. 2015 [27]	30	75	8.89	Hip fractures (low energy)	24	6
Johansson 2016 [28]	40	68	8.6	Proximal humeral fracture	40	0
Song et al. 2012 [35]	14	76.2	8.2	Femoral comminuted fracture	12	2
Peichl et al. 2011 [30]	65	82.3	4.1	Pelvic fracture	65	0
Aspenberg et al. 2010 [31]	102	61.4	8.6	Distal radius fracture	102	0

F, female; M, male.

**Table 2:** Detail of intervention.

Studies	Intervention		*N*_*e*_	*N*_*c*_	Treatment time	Time of initiation
	EG	CG				
Kanakaris et al. 2015 [27]	Teriparatide 20 *μ*g	Alendronate	9	21	4 weeks	—
		70 mg or vitamin D and calcium				

Johansson 2016 [28]	Teriparatide 20 *μ*g; analgesics	Analgesics	20	20	4 weeks	<10 days
	Physiotherapy	Physiotherapy				

Song et al. 2012 [35]	Teriparatide 20 *μ*g	No therapy	7	7	3 months	—

Peichl et al. 2011 [30]	PTH1–84 100 *μ*g; calcium 1000 mg	Calcium 1000 mg	21	44	24 months	<2 days
	Vitamin D 800 IU	Vitamin D 800 IU				

Aspenberg et al. 2010 [31]	Teriparatide	Placebo	68	34	8 weeks	<10 days
	20 or 40 *μ*g					

*N*_*e*_: number in experimental group, *N*_*c*_: number in control group.

**Table 3:** Sensitivity analyses based on various exclusion criteria for fracture healing time.

Excluded trial	Number of trials	Number of patients	EG	CG	MD (95% CI)	*P* value for MD	*I* ^2^, %	*P* value for heterogeneity
Aspenberg et al. 2010 [31]	2 [30, 35]	79	28	51	−6.24 [−7.20, −5.29]	<0.000001	0	0.7
Song et al. 2012 [35]	2 [30, 31]	147	76	71	−3.60 [−8.70, 1.49]	0.17	98	<0.000001
Peichl et al. 2011 [30]	2 [31, 35]	96	62	34	−3.66 [−9.59, 2.27]	0.23	86	0.008

EG, experimental group; CG, control group.

**Table 4:** Sensitivity analyses based on various exclusion criteria for functional outcome.

Excluded trial	Number of trials	Number of patients	EG	CG	MD (95% CI)	*P* value for MD	*I* ^2^, %	*P* value for heterogeneity
Aspenberg et al. 2010 [31]	3 [27, 28, 30]	134	49	85	−0.80 [−1.87, 0.27]	0.14	87	0.0005
Johansson 2016 [28]	3 [27, 30, 31]	186	91	95	−1.18 [−2.14, −0.22]	0.02	86	0.0008
Kanakaris et al. 2015 [27]	3 [28, 30, 31]	195	101	94	−1.32 [−2.06, −0.58]	0.0005	80	0.008
Peichl et al. 2011 [30]	3 [27, 28, 31]	160	89	71	−0.74 [−1.69, 0.21]	0.13	85	0.001

EG, experimental group; CG, control group.

**Table 5:** The GRADE evidence quality for each outcome.

Interventions for [condition] in [population]						
Outcomes intervention and comparison intervention	Illustrative comparative risks (95% CI)		Relative effect (95% CI)	Number of participants (studies)	Quality of the evidence (GRADE)	Comments
	Assumed risk *With comparator*	Corresponding risk *With intervention*				

*Fracture healing time*						
Teriparatide/fracture healing	The mean fracture healing time in the control groups was *9.1–23 weeks*	The mean fracture healing time in the intervention groups was *4.54 lower* (8.8 to 0.28 lower)		157 (3 studies)	⊕⊕*⊖⊖* low	

*Functional outcome*						
Teriparatide/fracture healing	The mean functional outcome in the control groups was *25.8–56.3 points*	The mean functional outcome in the intervention groups was *1.02 standard deviations lower* (1.81 to 0.22 lower)		225 (4 studies)	⊕⊕*⊖⊖* low	
